# Arterial blood gases predict long-term prognosis in stage I non-small cell lung cancer patients

**DOI:** 10.1186/s12893-016-0119-4

**Published:** 2016-01-13

**Authors:** Shinjiro Mizuguchi, Takashi Iwata, Nobuhiro Izumi, Takuma Tsukioka, Shoji Hanada, Hiroaki Komatsu, Noritoshi Nishiyama

**Affiliations:** Department of Thoracic Surgery, Osaka City University Hospital, 1-4-3 Asahimachi, Abeno-ku, Osaka 545-8585 Japan

**Keywords:** Arterial blood gas, Prognosis, Lung cancer, Early stage

## Abstract

**Background:**

Preoperative hypercapnia and hypoxemia are reportedly risk factors for postoperative complications. This study aimed to establish the long-term survival risk associated with abnormal preoperative arterial blood gases (ABGs) in patients with non-small cell lung cancer (NSCLC).

**Methods:**

This study involved 414 patients with stage I NSCLC who underwent lobectomy/bilobectomy with mediastinal lymph node dissection. The patients were divided into groups with normal (*n* = 269) and abnormal (*n* = 145) ABGs.

**Results:**

The patients in the normal ABG group (median age 67 years) were significantly younger than those in the abnormal ABG group (median age 70 years). There were no significant differences between the groups in gender, performance status, pathological stage, histology, postoperative complications, or preoperative comorbidity, except for chronic obstructive pulmonary disease/pulmonary fibrosis. The 3-, 5- and 10-year survival rates in the normal and abnormal ABG groups were 87, 77 and 56, and 78 , 63 and 42 %, respectively (*p* = 0.006). According to multivariate analysis, age, gender, performance status, non-adenocarcinoma, differentiation of resected tumor, pathological stage, any prior tumor and abnormal ABGs (risk ratio, 1.61) were independent prognostic factors.

**Conclusions:**

Abnormal ABGs predict long-term survival risk in patients with NSCLC, which is important for planning therapeutic strategies.

## Background

Surgery is the most effective therapy for early non-small cell lung cancer (NSCLC). Indeed, the 5-year survival rates in patients who underwent pulmonary resection were as follows: clinical stage IA, 82.0 % (*n* = 6295); pathological stage IA 86.8 % (*n* = 4978); clinical stage IB, 66.8 % (*n* = 2339); and pathological stage IB 73.9 % (*n* = 2552) [1]. The 5- and 10-year survival rates of 799 patients with screening-detected clinical stage I lung cancer who did not undergo surgical treatment were only 16.6 and 7.4 %, respectively [2]. A recent study of Japanese patients with operable cancer who elected to undergo stereotactic ablative radiotherapy reported 5-year overall survival rates of 72 and 62 % for stage IA and IB subgroups, respectively [3]. Therefore, anatomical lobectomy is performed in most cases, which is acceptable from a physiological point of view.

Prior to surgical treatment, oncological and physiological tolerance of the proposed procedure should be considered. To assess physiological tolerance, cardiopulmonary function evaluation, including general respiratory function tests, arterial blood gas (ABG) analysis, electrocardiography, and echocardiography, is important according to two recent sets of guidelines [4, 5]. According to a review of preoperative evaluation of lung cancer resection candidates, active smoking, dyspnea, age, proposed extent of surgery, and presence of vascular disease, diabetes mellitus and dysrhythmia are variably associated with increased risks of complications and/or mortality [6]. One study reported that 37 % of individuals who present with anatomically resectable disease are deemed unsuitable for surgery on the basis of poor pulmonary function alone [7]. Moreover, physiological variables have recently been shown to influence not only perioperative outcomes, but also long-term quality of life and survival after pulmonary resection in patients with NSCLC. Quality of life, particularly as it is related to physical function, such as forced expiratory volume in 1 s (FEV_1.0_) and diffusion capacity of the lungs for CO (DL_CO_) [8–10], is affected after surgical resection.

ABG tests have also been used to evaluate pulmonary function before thoracic surgery to prevent subsequent respiratory insufficiency. Historically, hypercapnia (partial pressure of CO_2_ in arterial blood [PaCO_2_] >45 Torr) has been considered an exclusion criterion for pulmonary resection [11, 12]. Hypercapnia and hypoxemia (<60 Torr partial pressure of O_2_ in arterial blood [PaO_2_] or <90 % oxygen saturation [SaO_2_]) are reportedly risk factors for postoperative complications [5]. However, according to British Thoracic Society guidelines, hypercapnia alone is not a predictor of complications after lung resection. Such patients are often precluded because of other adverse factors, such as postoperative FEV_1.0_ and DL_CO_ <40 % [13].

It has not been reliably established whether preoperative abnormal ABGs, such as low oxygenation, hypercapnia or hypocapnia, are predictors of prognosis in patients who undergo pulmonary resection. The hypothesis of this study is that abnormal ABG parameters are independently associated with a higher risk of long-term mortality, especially non-cancer related mortality. Given that the outcomes are not biased by organ failure caused by tumor compression or involvement, this study aimed to examine the correlation between preoperative ABG analysis and long-term risk of death in patients with stage I NSCLC.

## Methods

### Patients

The medical records of 587 continuous patients who underwent pulmonary resection for pathological stage I NSCLC at Osaka City University Hospital, from January 1998 to December 2012, were analyzed retrospectively. The following patients were excluded from the analysis: those who underwent partial/wedge resection, without radical mediastinal lymph node dissection (R0) as previously described [14], and those who underwent neoadjuvant therapy for lung cancer. Ultimately, the records of 414 patients with pathological stage I NSCLC who underwent lobectomy/bilobectomy with R0 were examined. Surgery was performed through an axillary mini-thoracotomy (assisted by video) or posterolateral thoracotomy (when extended lobectomy, e.g., chest wall resection, pulmonary arterioplasty or bronchoplasty was necessary). Histological classification was performed according to the World Health Organization criteria for histological typing of lung tumors [15]. Postoperative staging was based on the international TNM classification for lung cancer [16, 17]. Patients were followed at 1–6-month intervals postoperatively. Follow-up evaluation included physical examination, chest X-ray, and blood examination, including for tumor markers. Chest, brain and abdominal computed tomography were performed at 3 − 6-month intervals. Bone scanning was not routinely performed in asymptomatic patients. Whenever any symptoms or signs of recurrence were detected, magnetic resonance imaging of the brain and bone scintigraphy was performed. The patients (n = 53 /191; 28 %) with stage 1A (T1b) and 1B adenocarcinoma received oral fluoropyrimidine for 2 years. Dose escalation or schedule modification occurred at the discretion of the clinician. Patients underwent chemotherapy, radiotherapy, or the best available supportive care when recurrence was detected. The study was performed in accordance with the Declaration of Helsinki and received approval from the Institutional Review Board of the Osaka City University (Osaka, Japan, reference number 2793) with waiver of informed consent because of its retrospective design.

### Data collection and follow-up evaluation

The patients’ demographic data, surgical outcomes, and complications were reviewed, and the survival rate was analyzed. Follow-up results and causes of death were obtained from patients’ medical records (in our institute and with their practitioners/pulmonologists) and telephone calls, and the last follow-up day was June 30, 2015.

In-hospital mortality included all deaths during the first 30 days or postoperative hospital stay. Postoperative complications were classified as major (potentially life-threatening) or minor (not life-threatening) occurring within 30 days after surgery or bronchopleural fistula within 3 months. Prolonged air leakage was defined as persisting for >5 days after surgery. Prolonged pleural effusion was defined as >1.5 L until 5 days after surgery. Either or both predicted postoperative FEV_1.0_ and DL_CO_ <40 % were defined as high-risk comorbidity. Pulmonary assessment included complete history, physical examination, and pulmonary function tests (PFTs). Predicted postoperative lung function was calculated by the segments method based on the total number of segments (42), then corrected for segments that were obstructed by tumor preoperatively [18]. In our physiological exclusion criteria, postoperative FEV_1.0_ ≥ 600 mL was considered a prerequisite for resection. However, PaCO_2_ ≤ 50 Torr was considered a prerequisite for resection traditionally, but we did not exclude patients with only hypercapnia. This study included three patients with PaCO_2_ > 50 Torr (50.6, 50.3 and 50.2 Torr, respectively) and all patients had postoperative FEV_1.0_ ≥ 600 mL.

### ABG

The patients were divided into two groups according to the results of ABG analysis: a normal ABG group (*n* = 269) and an abnormal ABG group (*n* = 145) from a previous report [19] and our institution. The abnormal group included at least one of the following parameters: [1] PaO_2_ ≤ 80 Torr (*n* = 83, median 75, range 62–79; [2] PaCO_2_ ≤ 35 Torr (*n* = 22, median 34.0, range 31.1–34.9) or ≥45 Torr (*n* = 50, median 46.1, range, 45.0–50.6); or [3] pH ≤7.35 (*n* = 5, median 7.335, range 7.332–7.349), or ≥7.45 (*n* = 14, median 7.456, range 7.451–7.485).

### Statistical analysis

Comparison of continuous and dichotomous variables between the two groups was performed using the Mann − Whitney U test, the χ^2^ test, or Fisher’s exact test. The Kaplan − Meier method and log-rank test were used for survival analysis. Multivariate analysis was performed with a Cox regression model with forward stepwise selection and multivariate risk ratios with 95 % confidence intervals (CIs) were calculated. *P* < 0.05 was considered statistically significant. Statistical analysis was performed using JMP 10 software (SAS Institute, Cary, NC, USA).

## Results

### Clinicopathological features

Of these 414 patients, 302 had adenocarcinoma, 99 squamous cell carcinoma, eight adenosquamous carcinoma, and five large cell carcinoma. As to pathological stage, 232 patients were in stage IA and 182 in stage IB. Table 1 shows the clinicopathological characteristics of the two groups. The patients in the normal ABG group (median age 67 years) were significantly younger than those in the abnormal ABG group (median age 70 years) (*p* = 0.005, Mann − Whitney U test). There were no significant differences in gender, body mass index, smoking history, Eastern Cooperative Oncology Group performance status (PS), Hugh–Jones (H–J) classification, postoperative predicted PFTs, histology and differentiation of the resected tumor, pathological stage, tumor location, surgical procedures, blood loss, operative time, and adjuvant therapy between the two groups.

**Table 1:** Clinicopathological characteristics according to normal or abnormal ABGs

Group		Patients	Normal ABG	Abnormal ABG	*p* value
			(*n* = 269)	(*n* = 145)	
Age (years)	<70	233	164	69	0.009
	≥70	181	105	76	
Gender	Male	250	159	91	0.469
	Female	164	110	54	
BMI (Kg/m^2^)			22.2 ± 3.2	22.0 ± 3.4	0.539
Smoking History	Yes	261	165	96	0.326
	No	153	104	49	
PS	0-1	384	250	134	0.844
	2	30	19	11	
H–J classification	1-2	401	261	140	0.792
	3	13	8	5	
Predicted post PFTs^a)^	≥40	383	253	130	0.105
	<40	31	16	15	
Histology	Ad	302	203	99	0.326
	Sq	99	57	42	
	Others	13	9	4	
Differentiation	Well	133	87	46	0.898
	Mod/poor	281	182	99	
p-stage	IA	233	160	73	0.068
	IB	181	109	72	
Location	Rt. upper	133	89	44	0.815
	Rt. middle	28	17	11	
	Rt. lower	86	59	27	
	Lt. upper	92	57	35	
	Lt. lower	74	46	28	
Surgical procedure	Lobectomy	405	265	140	0.197
	Bilobectomy	9	4	5	
Blood loss (g)			125 ± 141	137 ± 143	0.234
Operative time (min)			192 ± 65	203 ± 67	0.483
Adjuvant therapy	Yes	53	29	24	0.099
	No	361	240	121	

*ad* adenocarcinoma; *BMI* body mass index; *p-stage* pathological stage; *sq* squamous carcinoma

^a^Predicted postoperative values of FEV_1.0_ or DL_CO_ <40 % are defined as high-risk results of pulmonary function tests

### Preoperative comorbidity and postoperative complications

The postoperative course was uneventful in 281 patients and one hospital death was associated with acute exacerbation of interstitial pneumonia (IP). As shown in Table 2, there were no significant differences in preoperative comorbidity, such as any prior tumor, hypertension, diabetes mellitus, and cerebral, cardiac, renal and liver diseases, between the normal and abnormal ABG groups. The patients in the abnormal ABG group had a higher ratio of pulmonary comorbidity (chronic obstructive pulmonary disease [COPD]/IP) compared with those in the normal ABG group.

**Table 2:** Preoperative comorbidity and postoperative complications according to normal or abnormal ABGs

Group	Patients	Normal ABG	Abnormal ABG	*p* value
		(*n* = 269)	(*n* = 145)	
Preoperative comorbidities				
Hypertension	105	63	42	0.216
Diabetes mellitus	61	37	24	0.444
Cerebral disease	36	20	16	0.215
Cardiovascular disease	53	32	21	0.459
COPD/IP	64	32	32	0.006
Moderate-to-sever renal disease^a)^	84	55	29	0.914
Child-Pugh classification (B or C)	9	7	2	0.416
Any prior tumor	96	63	33	0.879
Postoperative complications				
In-hospital mortality	1	0	1	
Major complications	16	10	6	0.832
Re-exploration/IVR	2	1	1	
Bronchopleural fistula	0	0	0	
Cerebral infarction	1	1	0	
Respiratory failure/pneumonia	9	5	4	
Other organ failure^b)^	4	3	1	
Minor complications	117	83	34	0.110
Continuous air leakage	50	34	16	0.633
Prolonged pleural effusion/chylothorax	31	21	10	0.737
Supraventricular arrhythmias	27	20	7	0.820
Atelectasis/obstructive symptoms	12	9	3	0.461
Wound/intrathoracic infection	11	7	4	0.925
Change in mental status	8	7	1	0.178
Paresis of recurrent laryngeal nerve	4	2	2	0.528

*IVR*, inverse ratio ventilation

^a^Moderate-to-severe renal disease was defined as estimated glomerular filtration rate <60 mL/min/1.73 m^2^

^b^Including cardiac, renal and liver failure

There were 16 major and 117 minor complications in the remaining 133 patients. The 16 major complications comprised: postoperative bleeding that required re-exploration/interventional radiology in two patients; cerebral infarction in one; respiratory failure/pneumonia that required mechanical ventilation in nine (including one with acute exacerbation of interstitial pneumonia previously described); and other organ failure in four. Minor complications occurred in 117 patients. Prolonged air leakage occurred in 50 (12.1 %) and supraventricular arrhythmia in 27 (5.8 %). There were no significant differences in postoperative complications between the normal and abnormal ABG groups.

### Long-term postoperative outcome

**Fig. 1:**
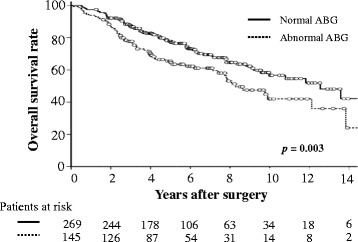
Survival rates of patients with resected stage 1 NSCLC according to preoperative ABG values. The survival of patients with abnormal ABG is significantly shorter than that in patients with normal ABG.

**Fig. 2:**
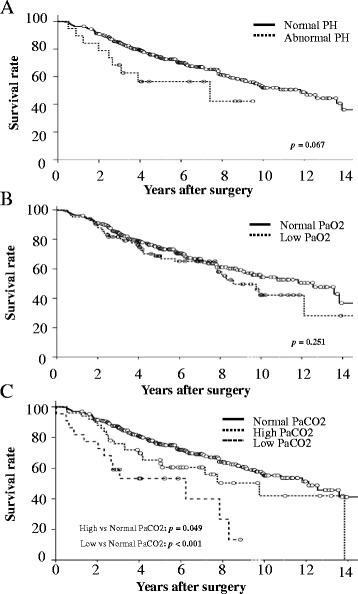
Overall survival of patients with stage 1 NSCLC according to preoperative ABG values: (**a**) pH, (**b**) PaO_2_ and (**c**) PaCO_2_

Although ten patients (2.4 %) were lost during follow-up, no patient was lost for ≥2 years. The overall mean duration of follow-up was 5.6 years (range 0.1–16.2 years) and 2343 patient-years were detected. There were 65 deaths from lung cancer, 80 from other diseases, including 23 related to other cancers, and 269 patients were still alive, including 16 with lung cancer recurrence. In the normal ABG group (*n* = 269), there were 35 deaths from lung cancer (13 %), 47 from other diseases (17 %) and 187 patients were still alive (70 %), whereas in the abnormal ABG group (*n* = 145), there were 30 deaths from cancer (21 %), 33 from other diseases (23 %) and 82 patients were still alive (56 %). As shown in Fig. 1, the 3-, 5- and 10-year survival rates in the normal and abnormal ABG groups were 87, 77 and 56 %, and 78, 63 and 42 %, respectively (*p* = 0.006). As shown in Fig. 2, there were no significant differences in survival between those with normal versus abnormal pH (*p* = 0.067), and normal versus low PaO_2_ (<80 Torr) (*p* = 0.251). The survival rates in the high (*p* = 0.049) and low (*p* <0.001) PaCO_2_ groups were significantly lower than those in the normal PaCO_2_ group.

**Fig. 3:**
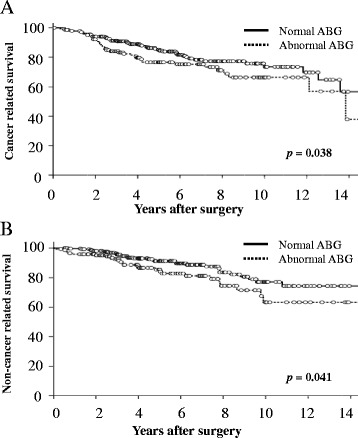
Overall survival based on cause of death in patients with stage 1 NSCLC according to preoperative ABG values: (**a**) all-cancer-related and (**b**) non-cancer-related survival rates

Although there were no significant differences between the two groups for cause of death (*p* = 0.554), the all-cancer-related (including secondary or any prior tumors) and non-cancer-related survival rates in the abnormal ABG group were significantly poor compared with those in the normal group (*p* = 0.038 and 0.041, respectively) as shown in Fig. 3.

### Multivariate analysis of ABG and clinical functions

According to the log-rank test, gender, age, PS (0–1 vs 2), ABG, postoperative predicted PFTs, histology, degree of tumor differentiation, pathological stage, postoperative complications and some preoperative comorbidity (cardiac, cerebral and liver disease and any prior tumor history) were significantly associated with survival, as shown in Table 3. According to multivariate analysis applying the above significant variables, gender, age, PS, histology (non-adenocarcinoma), differentiation, pathological stage, any prior tumor and ABG (risk ratio, 1.61; *p* = 0.006) were independent predictors of overall survival. When multivariate analysis was performed using ABG variables, both high PaCO_2_ (risk ratio, 1.84; *p* = 0.016) and low PaCO_2_ (risk ratio, 1.99; *p* = 0.043) were identified as independent factors.

**Table 3:** Results of survival analysis

Factors	Univariate	Multivariate	Risk ratio	95 % CI
	(*p* value)	(*p* value)		
Gender (Male vs Female)	<0.001	<0.001	2.40	1.55–3.81
Age (≥70 years)	<0.001	<0.001	2.55	1.78–3.69
PS (2 vs. 0–1)	<0.001	0.036	1.85	1.04–3.09
H–J classification (3 vs. 1–2)	0.070	–	–	–
Smoking	0.001	0.487	–	–
ABG (Abnormal vs. Normal)	0.002	0.006	1.61	1.14–2.26
Predicted post PFT^a)^	0.016	0.059	–	–
Histology (others vs. Ad)	0.017	0.011	1.65	1.12–2.44
Differentiation (m/p vs well)	<0.001	0.014	1.71	1.11–2.71
p-Stage (IB vs IA)	<0.001	0.002	1.74	1.23–2.48
Operative procedures^b)^	0.470	–	–	–
Location	0.268	–	–	–
Postoperative complication	0.017	0.320	–	–
Adjuvant therapy	0.629	–	–	–
Preoperative comorbidities				
Hypertension	0.533	–	–	–
Diabetes mellitus	0.652	–	–	–
eGFR (<60 ml/min/1.73 m^2^)	0.078	–	–	–
Child-Pugh classification (B or C)	0.031	0.389	–	–
Cardiac disease	0.011	0.241	–	–
Cerebral disease	0.010	0.594	–	–
Any prior tumors	<0.001	<0.001	3.49	2.37—5.10

*eGFR* estimated glomerular filtration rate; *m/p* moderate or poorly

^a^Predicted postoperative values of FEV_1.0_ or DLco <40 % is defined as high-risk for PFTs

^b^Operative procedures, lobectomy versus more than lobectomy

## Discussion

The key finding of this study is that preoperative ABG is a predictor of long-term prognosis in stage I NSCLC, independent of other important determinants such as age, gender, PS and pathological stage. H–J classification, predicted postoperative PFTs, and cardiac/cerebral disease, which are considered important physiological factors, were not independent prognostic factors according to multivariate analysis.

To select a subgroup of patients with stage I disease that might benefit from adjuvant therapy, many investigators have attempted to identify pathological prognostic factors. These attempts have included analysis of histological subtype, size of primary tumor, degree of tumor differentiation, tumor markers, and lymphatic or vascular invasion [20, 21]. Recent studies have reported that the following molecular markers are associated with poor prognosis or recurrence in stage I NSCLC: cell cycle regulation/apoptosis, angiogenesis, growth regulation, cellular adhesion, cell cycle regulation, and basement membrane invasion [20, 22]. As for physiological factors, age, gender, PS, weight loss, depressed mood, quality of life, smoking [20], Charlson Comorbidity Index score [23], FEV_1.0_/DL_CO_ [8–10] have been reported as long-term prognostic factors in patients undergoing pulmonary resection. The Charlson Comorbidity Index allots weighted scores based on the relative mortality risk to 19 factors that were found to significantly influence survival in the study subjects [23, 24]. Although these 19 factors include some such as heart failure, chronic pulmonary disease and renal disease that could affect ABG, abnormal ABG was not identified as a prognostic comorbidity factor in that study.

The preoperative physiological assessment of a patient being considered for surgical resection of lung cancer must take into account the immediate perioperative risks from comorbid cardiopulmonary disease, the long-term risks of pulmonary disability, and the threat to survival posed by inadequately treated lung cancer. As is evident in recently published algorithms, selection based on physiological variables of patients for major pulmonary resection for NSCLC currently focuses on perioperative outcomes [5]. Many authors have assessed physiological prognostic factors in patients with NSCLC and focused on PFTs, mainly preoperative or predicted postoperative FEV_1_ and DL_CO_ [7–10, 25–27]. Predicted postoperative pulmonary function can be theoretically determined by the amount of parenchymal resection. However, various factors can also influence it, including site of resection (upper or lower lobectomy), severity of pulmonary emphysema/fibrosis, the surgical approach (open or video-assisted thoracic surgery), and chemotherapy/radiation therapy [26]. When multivariate analysis was performed excluding ABG, predicted postoperative FEV_1.0_ and DL_CO_ (shown as predicted PFT) was an independent prognostic marker (risk ratio, 1.79, 95 % CI, 1.01–3.02; *p* = 0.047) in this study.

Abnormal ABG in patients with lung cancer undergoing surgical resection is reportedly a comorbidity risk factor (mainly respiratory disorder) [5, 11, 12]. However, this is controversial because two series have reported that perioperative complications are not more numerous in patients with preoperative hypercapnia [25, 28]. In the present study, we also found no significant difference in postoperative complications, including respiratory failure, between patients with preoperative CO_2_ in the normal range and those with hypercapnia. When multivariate analysis was performed using three ABG variables as for physiological factors, both high and low PaCO_2_ were identified as independent factors and further, larger studies are necessary to clarify these findings.

We did not routinely measure postoperative ABG in patients without complications, therefore preoperative or short-term changes in PaO_2_ or PaCO_2_ are unclear. In a previous study, although small decreases in PaO_2_ were detected after pneumonectomy, there were no significant decreases in PaO_2_ after minor resection (including lobectomy) and no significant changes in PaCO_2_ after any type of resection [27]. Low PaO_2_ (<80 Torr) was not a comorbidity predictor of prognosis in patients with early lung cancer undergoing pulmonary resection (Fig. 2b, *p* = 0.251). This may be of interest regarding home oxygen therapy.

We postulated that patients with abnormal ABGs might have poor prognosis because of the increased risk of non-cancer-specific death. It is important to note that deaths from both cancer and other causes were higher in the abnormal ABG group. It is possible that abnormal ABGs might affect or be affected by cancer biology, and further, larger studies are necessary to clarify our findings and determine whether this trend is significant.

## Conclusion

In the present study, we found that preoperative ABGs, not only hypercapnia but also hypocapnia are predictors of long-term prognosis in stage I NSCLC. Surgical strategies for patients with abnormal ABG values should be planned considering not only immediate or short-term risk, which relates to perioperative morbidity and mortality, but also long-term survival risk.

## Abbreviations

ABG: arterial blood gas
DL_CO_: diffusion capacity of the lungs for CO
FEV_1.0_: forced expiratory volume in 1 second
H–J classification: Hugh–Jones classification
NSCLC: non-small cell lung cancer
PaCO_2_: partial pressure of CO_2_ in arterial blood
PaO_2_: partial pressure of oxygen in arterial blood
PFTs: pulmonary function tests
PS: performance status
SaO_2_: oxygen saturation
